# Neuroimaging Evidence of Major Morpho-Anatomical and Functional Abnormalities in the BTBR T+TF/J Mouse Model of Autism

**DOI:** 10.1371/journal.pone.0076655

**Published:** 2013-10-16

**Authors:** Luca Dodero, Mario Damiano, Alberto Galbusera, Angelo Bifone, Sotirios A. Tsaftsaris, Maria Luisa Scattoni, Alessandro Gozzi

**Affiliations:** 1 Istituto Italiano di Tecnologia, Center for Neuroscience and Cognitive Systems @UniTn, Rovereto, Italy; 2 IMT - Institute for Advanced Studies Lucca, Italy; 3 Department of Electrical Engineering and Computer Science, Evanston, Illinois, United States of America; 4 Istituto Superiore di Sanità, Neurotoxicology and Neuroendocrinology Section, Department of Cell Biology and Neurosciences, Rome, Italy; Tulane University Medical School, United States of America

## Abstract

BTBR T+tf/J (BTBR) mice display prominent behavioural deficits analogous to the defining symptoms of autism, a feature that has prompted a widespread use of the model in preclinical autism research. Because neuro-behavioural traits are described with respect to reference populations, multiple investigators have examined and described the behaviour of BTBR mice against that exhibited by C57BL/6J (B6), a mouse line characterised by high sociability and low self-grooming. In an attempt to probe the translational relevance of this comparison for autism research, we used Magnetic Resonance Imaging (MRI) to map in both strain multiple morpho-anatomical and functional neuroimaging readouts that have been extensively used in patient populations. Diffusion tensor tractography confirmed previous reports of callosal agenesis and lack of hippocampal commissure in BTBR mice, and revealed a concomitant rostro-caudal reorganisation of major cortical white matter bundles. Intact inter-hemispheric tracts were found in the anterior commissure, ventro-medial thalamus, and in a strain-specific white matter formation located above the third ventricle. BTBR also exhibited decreased fronto-cortical, occipital and thalamic gray matter volume and widespread reductions in cortical thickness with respect to control B6 mice. Foci of increased gray matter volume and thickness were observed in the medial prefrontal and insular cortex. Mapping of resting-state brain activity using cerebral blood volume weighted fMRI revealed reduced cortico-thalamic function together with foci of increased activity in the hypothalamus and dorsal hippocampus of BTBR mice. Collectively, our results show pronounced functional and structural abnormalities in the brain of BTBR mice with respect to control B6 mice. The large and widespread white and gray matter abnormalities observed do not appear to be representative of the neuroanatomical alterations typically observed in autistic patients. The presence of reduced fronto-cortical metabolism is of potential translational relevance, as this feature recapitulates previously-reported clinical observations.

## Introduction

Autism spectrum disorder (ASD) is a heterogeneous group of neuro-developmental conditions of uncertain etio-pathology characterised by pronounced social and cognitive deficits. The results of multiple neuroimaging studies are converging to suggest the presence of shared patterns of structural and functional alterations in the brain of autistic patients. For example, structural Magnetic Resonance Imaging (MRI) has consistently highlighted reduced volume of amygdala, hippocampus, corpus callosum and parietal regions in children and adult ASD patients [1], [2]. Similarly, Diffusion Tensor Imaging (DTI) assessments of white matter (WM) have highlighted decreased fractional anisotropy (FA), an index of organisation and coherence of WM tracts, in several brain regions of autistic patients compared to control groups [3], [4]. Abnormalities in resting-state brain function have also been described, including reduced fronto-cortical perfusion [5], [6] and metabolism [7], as well as a marked impairment in long-range functional connectivity [4].

While autism may be uniquely human, attempts to mimic symptoms and components of the disorder in animal models have been described [8]. The inbred BTBR T+tf/J (BTBR) strain has recently gained interest in the preclinical community because it displays robust analogies to all three of the diagnostic symptoms of autism, including deficits in reciprocal social interactions [9]–[12] impaired communication [12]–[14] and repetitive behaviours [e.g. repetitive self-grooming 15,16] as compared with high sociability and low self-grooming reference inbred lines such as C57BL/6J (B6) [12]–[14], [17]–[20]. These features have prompted the use of BTBR mice as a potential behavioural assay to evaluate novel pharmacological treatments for ASD [18], [21].

Recent neuroanatomical studies have highlighted major WM alterations in BTBR mice with respect to control B6 mice, with agenesis of the corpus callosum and lack of hippocampal commissure, a feature that may be partially compensated by a putative accessory inter-hemispheric WM tract [19], [22], [23]. The presence of major callosal alterations has been recently confirmed using FA mapping with MRI [24]. The same study also highlighted foci of altered brain and gray matter (GM) volume in cortical and subcortical areas of the BTBR mice [24]. These initial studies argue for the presence of significant neuroanatomical alterations in BTBR mice with respect to B6 controls. However, the translational relevance of such a cross-strain (e.g. BTBR vs B6) comparison for autism research remains undetermined.

In order to begin to address this question, we used MRI in BTBR and B6 mice to map morpho-anatomical and functional neuroimaging readouts that have been extensively used in patient populations. Specifically, DTI was used to map inter-group differences in FA using tract-based spatial statistics (TBSS) and to describe the three-dimensional topology of WM via the use of deterministic DTI tractography. Local GM volume alterations were mapped using two independent methods (voxel-based morphometry and cortical thickness mapping) widely employed in human neuroimaging studies. Finally, resting-state brain activity was assessed using cerebral blood volume weighted fMRI, an established marker of resting neuronal activity and metabolism in rodents [25]–[27] and humans [28]. By directly comparing anatomical and functional readouts of pathological relevance in men and mice, our findings permit an objectifiable assessment of the translational relevance of this animal model of autism, thus bridging clinical and preclinical research.

## Materials and Methods

### Ethical Statement

All in vivo studies were conducted in accordance with the Italian law (DL 116, 1992 Ministero della Sanità, Roma) and the recommendations in the Guide for the Care and Use of Laboratory Animals of the National Institutes of Health. Animal research protocols were also reviewed and consented to by the animal care committee of the Istituto Italiano di Tecnologia (permit 07-2012). All surgical procedures were performed under anaesthesia.

### Structural Magnetic Resonance Imaging

High-resolution morpho-anatomical T2-weighted and diffusion-weighted MR imaging of mouse brains was performed in adult (15–26 weeks) male BTBR T+tf/J (N = 9) and C57Bl/6 (N = 9) mice (Charles River Lab, Como, Italy). MRI imaging was performed in paraformaldehyde (4% PFA) fixed brains, a procedure that permits to obtain artefact-free high-resolution images devoid of physiological or motion artefacts [29]. Animals were anaesthetized with an intraperitoneal Avertin injection (375 mg/Kg) and perfused intracardially with PBS followed by paraformaldehyde (4% PFA). Both perfusion solutions were added with a Gadolinium chelate (Prohance, Bracco, Milan) at a concentration of 10 and 5 mM, respectively to reduce longitudinal relaxation times. Brains were imaged inside intact skulls to avoid post-extraction deformations.

MR images were acquired within 6 days from perfusion at 7.0 Tesla using a using a 72 mm birdcage transmit coil, and a custom-built saddle-shaped solenoid coil for signal reception with the following imaging parameters: 3D RARE spin-echo sequence, TR = 550 ms, TE = 33 ms, RARE factor = 8, echo spacing 11 ms and voxel size of 90 µm^3^(isotropic).

Diffusion tensor images (DTI) were acquired with 81 different gradient orientations at a b value of 1262 s/mm^2^ (∂ = 5 ms, Δ = 10 ms), in-plane spatial resolution of 130×130 µm^2^, and slice thickness of 350 µm in the coronal plane, using a 4-shot EPI sequence with TR = 5500 ms and TE = 26 ms, 20 averages for a total acquisition time of 10 hrs 52 m. For each DTI dataset, 8 co-centered volumes were acquired with no diffusion weighting (b = 0). Co-centered T2 weighted images were also acquired with the same resolution of the DTI volumes, using a 2-D fast spin-echo sequence.

### Tract-based Spatial Statistics of FA and DTI Tractography

Inter-group differences in spatial distribution and magnitude of DTI fractional anisotropy were mapped using tract-based spatial statistics (TBSS) as previously described [25], [30]. To this purpose, DTI images were re-sampled to obtain isotropic voxel dimensions (130 µm^3^) and non brain tissue was manually removed using b = 0 images as a reference. The binary mask thus obtained was used for subsequent skull stripping. Fractional anisotropy (FA) images were created by fitting a tensor model to the raw diffusion data using FSL [30].

A common FA space (FA template) including all the control subjects (B6, N = 9) of the study was created by non linearly-mapping FA data to a representative subject. The template was then used as reference for two rounds of linear and non-linear registrations of individual FA images using FSL’s FLIRT (affine) and FNIRT (non-linear) algorithms, respectively. The template was thinned at a threshold level of 0.3 to create a mean FA that represents the centres of the major FA tracts in the mouse brain. Each subject's aligned FA data were then projected onto this skeleton and the resulting data fed into voxel-wise cross-subject statistic as using a nonparametric permutation test with 5000 permutations (Nichols & Holmes, 2002). Data were thresholded at a Z level of 1.96 and corrected for multiple comparisons using a cluster-based threshold (pc) of 0.01. Mean FA in manually-drawn regions-of-interests (ROIs) was also computed for each subject based on anatomical correspondence with a stereotactic atlas of the mouse brain [31]. Inter-group statistical analysis of mean FA was carried out using a Student’s t-test, followed by Hochberg’s correction for multiple comparisons and a corrected level of statistical significance (pc) of 0.05. Diffusion Tensor Tractography (DTT) of BTBR and B6 control subjects was performed by estimating axonal fibres projections with a deterministic fibre assignment using the continuous tracking algorithm [32]. Criteria for terminating the tracking of individual fibres included an anisotropy threshold (values below 0.15) and a maximum stiffness condition, so that the tracking was terminated when the diffusion directions in consecutive steps differed by more than 35°. A multiple region of interest (ROI) approach to extract all the tracts of interest was used; the tracts of interest for each subject were linearly registered using the affine linear matrix obtained from previous registration to the FA template, so the properties of the tracts could quantified in the same space.

### Voxel based Morphometry (VBM)

Inter-group differences in local gray-matter volume were mapped using voxel-based morphometry (VBM) [25], [33]. A detailed description of the method can be found elsewhere [34]. Briefly, a study-specific template was created aligning high-resolution T2W images of the B6 control population (N = 9) to a common reference space via a 12 degrees-of-freedom (DOF) affine alignment, followed by 5 consecutive symmetric diffeomorphic registrations [35], [36].

Human VBM studies have demonstrated superior accuracy using study-based templates with respect to the use of prior reference atlases [37]–[39]. Moreover, it has been shown that the number of subjects used to create the reference study-template marginally influences detection accuracy, and that equally-robust findings can be using of varying numbers (N = 5–50) of randomly-chosen control subjects [37]. Individual T2W images of two groups of subjects were then nonlinearly registered to the study-based template using diffeomorphic registration. Visual examination of co-registered images did not highlight any regional displacement or disproportionality as a consequence of the lack of corpus callosum in BTBR mice. GM of spatially-normalized subjects was then segmented using a Markov Random Field model using a 6 class segmentation of the study-based template as a prior to initialize the process [40]. The Jacobian determinants of the deformation were then used to modulate the GM probability maps calculated during the segmentation step. The modulation compensates for the deformation introduced after the spatial normalization so that there is no variation of the total amount of GM, focusing the analysis on the local volumetric variation of the GM instead of the tissue density [33]. The resulting modulated GM probability maps were then smoothed using a gaussian kernel with a 3 voxels width sigma for voxel-wise statistical parametric comparison. Voxel-wise cross-subject statistic was performed using a nonparametric permutation test with 5000 permutations (Nichols & Holmes, 2002). Data were corrected for multiple comparisons using a cluster-based threshold (pc) of 0.01.

Mean GM volumes in manually-drawn regions-of-interests (ROIs) were also computed for each subject based on anatomical correspondence with a stereotactic atlas of the mouse brain [31]. Inter-group statistical analysis of mean GM volumes was carried out using a Student’s t-test, followed by Hochberg’s correction for multiple comparisons and a corrected level of statistical significance (pc) of 0.05.

### Cortical Thickness Mapping

A voxel-based method for the measurement of cortical thickness was developed by implementing the approach originally described by Das et al. [41], [42]. For each cortical hemisphere, the method identifies a continuous one-to-one correspondence between the inner and the outer cortical surface. Cortical territories were defined via label propagation as previously described [43]. The study-based template generated for VBM was registered non-linearly to an anatomically-parcellated high-resolution MRI atlas of the B6 mouse brain [44]. The cortical label of the template were then back propagated to the study-based template and to each subject’s space via diffeomorphic registrations [45]. Cortical thickness was then estimated on the basis of this diffeomorphic correspondence as described in [41]. In order to preserve Neumann boundary, the process was performed separately for each hemisphere [43] producing voxel-based thickness maps in the subject space. The maps were then re-transformed into study-template space and smoothed, to allow for voxel-level statistical analysis. Voxel-wise cross-subject statistics was performed using a nonparametric permutation test with 5000 permutations (Nichols & Holmes, 2002). Data were corrected for multiple comparisons using a cluster-based threshold (pc) of 0.01.

### Functional MRI

fMRI experiments were performed in adult (26 weeks) male BTBR T+tf/J (N = 10) and C57Bl/6 (N = 10) mice as previously described [46]–[48]. Briefly, mice were anaesthetized with isoflurane, intubated and artificially ventilated. The left femoral artery was cannulated for compound administration, continuous blood pressure monitoring, infusion of paralysing agent (gallamine thriethiodide, Sigma-Aldrich, 20 mg/kg/hr) and blood sampling for terminal measurement of arterial blood gases (p_a_CO2 and p_a_O2). Mean p_a_CO_2_ and p_a_O_2_ levels are reported in Table 1. No statistically significant inter-group difference in arterial blood gas levels was observed (p>.22, all comparisons, Student’s t-test).

**Table 1:** Post-MRI blood gas levels.

Group	p_a_CO_2_	p_a_O_2_
B6	25±1	283±22
BTBR	23±2	287±6

p_a_CO_2_ partial pressure of arterial CO_2_ measured at the end of the fMRI scan. p_a_CO_2_ partial pressure of arterial CO_2_ measured at the end of the fMRI scan. Arterial blood gas values are expressed in mmHg (mean ± SEM).

MRI data were acquired using a Bruker 7 Tesla scanner. Co-centred anatomical images and fMRI times series were acquired using a RARE and a Fast Low-Angle Shot (FLASH) MRI sequence, respectively, with the following imaging parameters: a) RARE TR = 3000 ms, effective TE = 38 ms, RARE factor 8, FOV 40 mm, 100×100×500 µm resolution b) FLASH: TR = 288 ms, TE = 3.1 ms, α = 30°; FOV 40 mm, 180×180×600 µm resolution, dt = 60 s, N = 30, corresponding to 30 min total acquisition time. Images were sensitized to reflect alterations in rCBV using 5 µl/g of blood-pool contrast agent (Molday Ion, Biopal Inc, Worcester, USA) which was injected after 5 baseline images.

The procedure used to calculate basal CBV (bCBV) has been recently described in great detail [25], [49]. Briefly, CBV-weighted time series were spatially normalized to a reference B6 anatomical template [50], and signal intensity was converted into basal cerebral blood volume (bCBV(t)) pixel-wise. bCBV time-series were calculated over a 5 minute time-window starting 15 min after contrast agent injection. Voxel-wise group statistics was carried out using FSL using multi-level Bayesian inference and a Z threshold >3.1 and a corrected cluster significance threshold (pc) of p = 0.01. Mean bCBV in manually-drawn regions-of-interests (ROIs) was also computed for each subject based on anatomical correspondence with a stereotactic atlas of the mouse brain [31]. Inter-group statistical analysis of mean GM volume was carried out using a Student’s t-test, followed by Hochberg’s correction for multiple comparisons and a corrected level of statistical significance (pc) of 0.05.

## Results

### Major White Matter Reorganization in BTBR Mice

**Figure 1:**
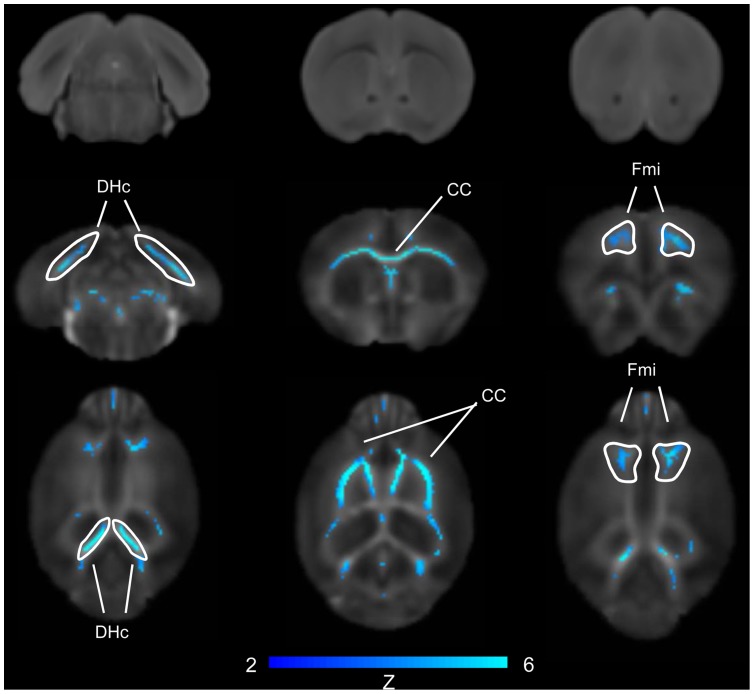
Major FA reduction in hippocampal commissure and corpus callosum of BTBR white matter. Voxelwise significant FA differences between BTBR (N = 9) versus B6 (N = 9, Z = 1.96, pc = .01). Representative T2-weighted anatomical images from a BTBR subject (top row) are shown for comparison. [CC: corpus callosum; DHc: dorsal hippocampal commissure; Fmi: Forceps minor of the corpus callosum].

Recent neuroanatomical studies have highlighted agenesis of the corpus callosum in BTBR mice, [19], [22], [23]. In order to probe the integrity of other inter-hemispheric connections and map the three-dimensional topology of WM in these animals, we first performed voxel-wise mapping of inter-group differences in fractional anisotropy using tract-based spatial-statistics (TBSS) and subsequently carried out deterministic WM tractography on the same DTI datasets. Areas of significantly reduced fractional anisotropy in BTBR mice were identified across the whole antero-posterior extension of the corpus callosum including its more frontal portions (forceps major) but also the dorsal hippocampal and anterior commissure (Z>2, pc = .01, Figure 1). The presence of marked reductions in FA in BTBR mice was apparent using a region-of-interest (ROI) approach (Figure S1). The effect was pronounced and statistically significant in the dorsal hippocampal commissure, and in the corpus callosum (p<0.01, Student’s t-test followed by Hochberg’s correction).

**Figure 2:**
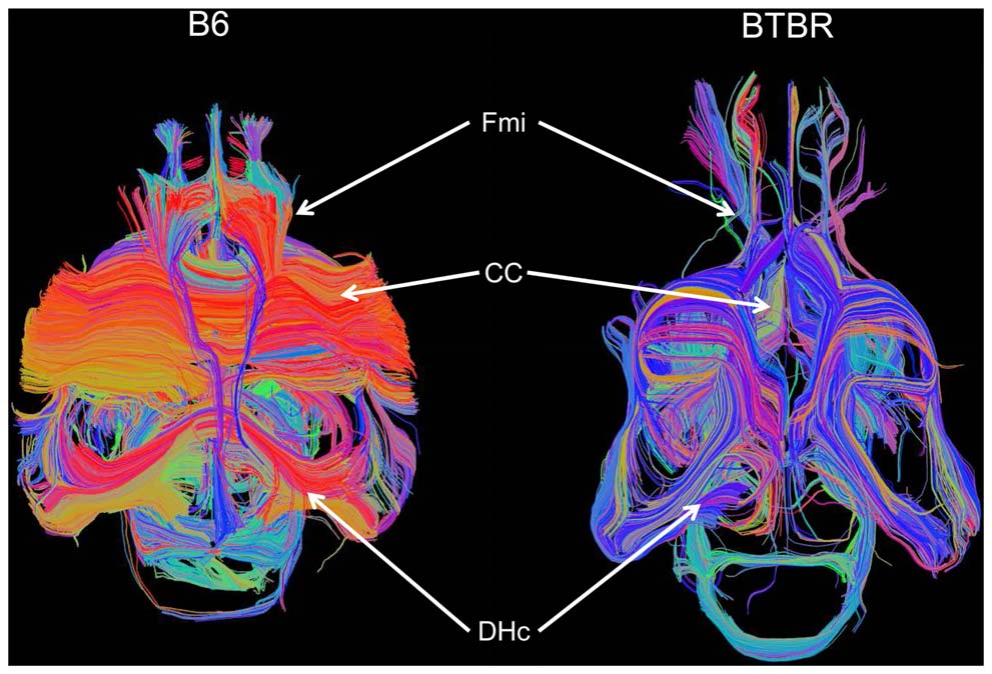
Major white matter reorganisation in BTBR mice. Diffusion tensor tractography of white matter in a representative B6 (left) and BTBR (right) subject. Only white matter tracts originating from regions exhibiting statistically significant FA alterations are displayed [CC: corpus callosum; DHc: dorsal hippocampal commissure; Fmi: Forceps minor of the corpus callosum].

**Figure 3:**
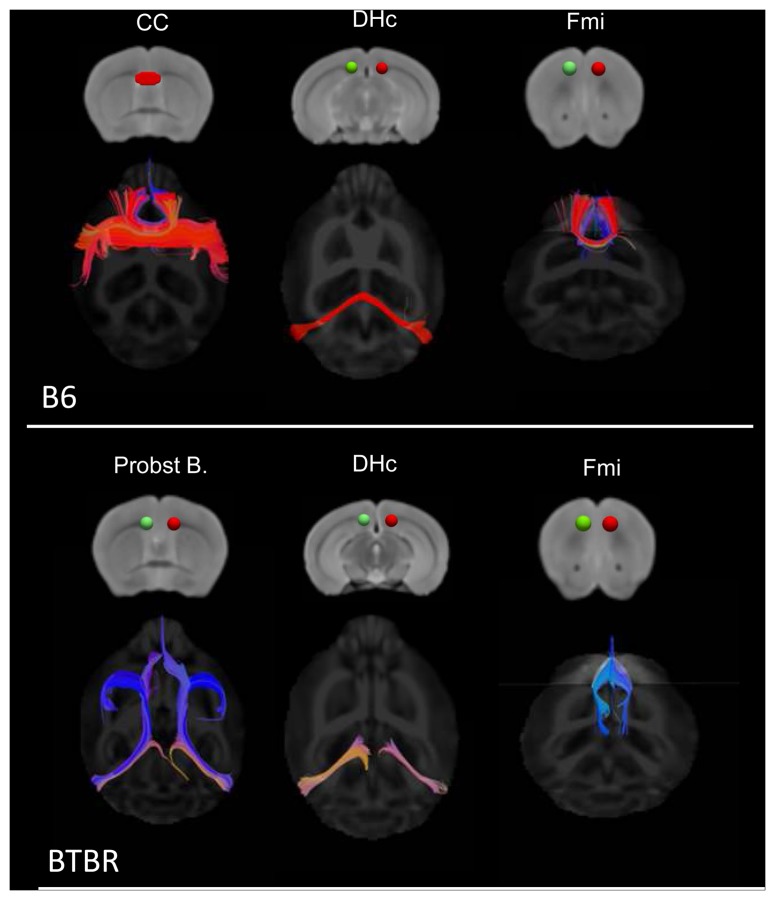
Antero-posterior white matter reorganisation in BTBR mice. Diffusion tensor tractography of representative dorsal inter-hemispheric tracts in control (top) and BTBR (bottom) mice. Seed regions used for tractography are displayed in green/red [CC: corpus callosum; DHc: dorsal hippocampal commissure; Fmi: Forceps minor of the corpus callosum; Probst B.: Probst bundles].

**Figure 4:**
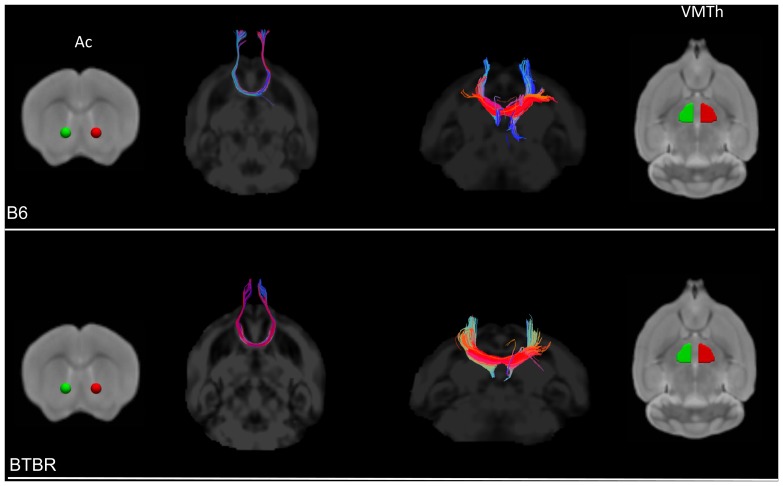
Intact ventral inter-hemispheric tracts in BTBR mice. Diffusion tensor tractography of representative ventral inter-hemispheric tracts in control (top) and BTBR (bottom) mice. Seed regions used for tractography are displayed in green/red [Ac: anterior commissure; VMTh: ventromedial thalamus].

To obtain a description of the overall spatial rearrangement of WM in BTBR mice, DTT tracts in representative BTBR and B6 subjects were created using as seed areas the regions exhibiting statistically-significant reductions in FA (Figure 2). Such representation highlighted the lack of inter-hemispheric connections in the corpus callosum and dorsal hippocampal commissure of BTBR subjects, together with a rostro-caudal reorganisation of WM tracts in these animals. These features were confirmed by subsequent DTT analyses using focal anatomical areas as seed regions (Figure 3), where all (N = 9) the BTBR subjects examined exhibited lack of inter-hemispheric tracts in the corpus callosum and hippocampal commissure. Moreover, tractography of Probst bundles, a barrel-shaped region of WM that forms where callosal interruptions take place [51], highlighted the presence of bilateral rostro-frontal WM fibre bundles in BTBR mice that were not present in any of the B6 controls examined (N = 9). Similarly, tracts originating from the forceps major of the corpus callosum in BTBR did not exhibit inter-hemispheric extension, but rather developed along the longitudinal axis of the brain. Intact inter-hemispheric WM bundles were identified in the anterior commissure and in ventro-medial thalamic regions in all the BTBR subjects examined (Figure 4).

A recent neuroanatomical study suggested the presence of a putative “accessory” inter-hemispheric WM tract that could partially-restore cortical inter-hemispheric connectivity in BTBR mice [23]. DTI tractography confirmed the presence of such a formation in 6 out of 9 BTBR subjects (Figure S2), where homotopic cortico-cortical inter-hemispheric fibres were found to innervate somatosensory regions of the posterior cortex via an indirect pathway that extended fronto-rostrally in the ventral portions of the brain. No homologue structure was found in any of the B6 subjects imaged.

### Reduced Cortical Thickness and Gray Matter Volume in BTBR Mice

**Figure 5:**
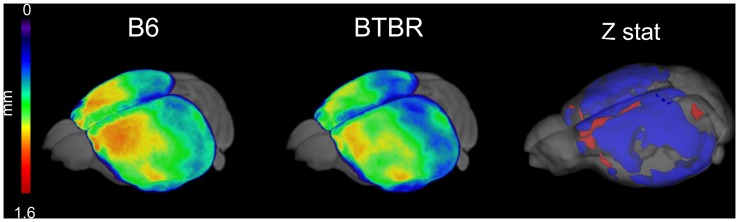
BTBR exhibit widespread cortical thickness reductions. Average cortical thickness in B6 (left) and BTBR mice (center) and corresponding voxel-wise statistics (right, Z>2, pc = .01; blue indicates BTBR<B6, red indicates BTBR>B6).

The presence of local alterations in GM volume in BTBR mice compared to B6 controls was investigated using cortical thickness mapping. A generalised reduction in mean cortical thickness (Z>1.96, pc = .01) was apparent in BTBR mice (Figure 5) in several cortical areas. Inter-group voxel-wise statistics revealed significant effects in fronto-occipital regions, parietal, temporal and peri-hippocampal cortex. Foci of increased cortical thickness were observed in the medial prefrontal and insular cortical regions (Z>1.96, pc = .01).

**Figure 6:**
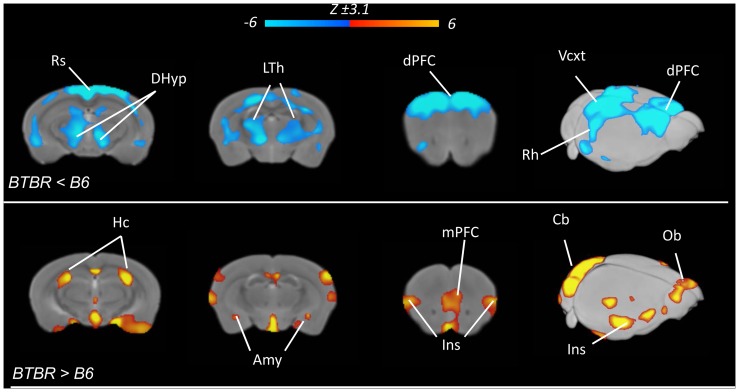
Widespread gray matter volume reductions in BTBR mice. Maps of the brain regions exhibiting altered grey matter volume in BTBR (N = 9) with respect to B6 controls (N = 9, Z>3.1, pc = .01). Blue indicates reduced, red/yellow indicates increased gray matter volume in BTBR mice vs B6 controls [Amy: amygdala; Cb: cerebellum; DHyp: dorsal hypothalamus; dPFC: dorsal prefrontal cortex; Ins: insular cortex; LTh: lateral thalamus; mPFC: medial prefrontal cortex; OB: olfactory bulbs; Rs: retrosplenial cortex; Vcxt: visual cortex].

The observation of prevalent cortical thickness alteration in BTBR mice prompted us to investigate the presence of GM alterations in non-cortical areas using whole-brain voxel based morphometry (VBM) of GM. In agreement with cortical thickness results, VBM analysis revealed widespread and bilateral reductions in GM volume across dorso-frontal, cingulate, retrosplenial, occipital and parietal cortex (Figure 6, Z>3.1, pc = .01). Among subcortical regions, major GM reductions were observed in the lateral and posterior thalamus (longitudinal fasciculus), and posterior hypothalamic regions. Foci of decreased volume were also observed in ventral-hippocampal areas. Focal increases in GM volume in BTBR were observed in the olfactory bulbs, medial pre-frontal and insular cortex, amygdala and dorsal hippocampal portions. ROI-based analysis corroborated these findings (Figure S3). Statistically-significant reductions in GM volume were observed in the dorsal hypothalamus, lateral thalamus, dorsal prefrontal, and retrosplenial cortex of BTBR mice, while increased GM volume was observed in the amygdala, dorsal hippocampus, and medial prefrontal cortex (p<0.01, all regions, Student’s t-test followed by Hochberg’s correction).

### Reduced basal Cortical Function in BTBR Mice

**Figure 7:**
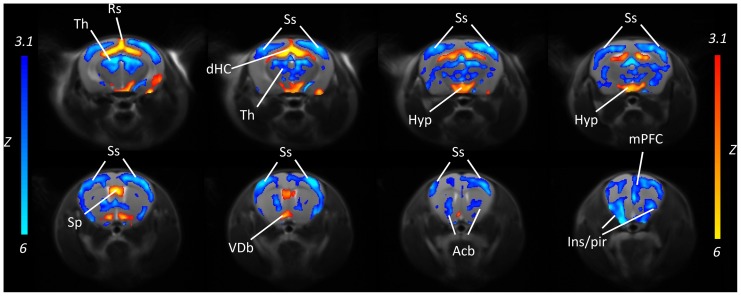
Reduced basal functional activity in the cortex of BTBR mice. Coronal view of the brain regions exhibiting increased (yellow-red) or reduced (blue) bCBV in BTBR (N = 10) versus B6 control subjects (N = 10, Z>3.1, pc = .01) [Acb: nucleus accumbens; Hyp: hypothalamus; Ins/Pir: insular/piriform cortex; mPFC: medial prefrontal cortex; Sp: septum; Ss: somatosensory cortex; Th; thalamus; Rs: retrosplenial cortex; VDb: ventral diagonal band].

In order to probe resting brain function in BTBR mice, we performed fMRI assessment of basal CBV (bCBV), an established marker of neuronal activity and metabolism [25], [49], [52], and mapped the regions exhibiting statistically significant differences (Z>3.1, pc = .01) in the two strains. A composite pattern of bCBV alterations was observed in BTBR with respect to B6 controls (Figure 7). Widespread bCBV reductions were found in cortical areas, with a prevalent involvement of medial-prefrontal areas, somatosensory and anterior insular and piriform regions. Foci of increased bCBV in BTBR mice were present in the ventral brain regions such the diagonal band and hypothalamus, and in the dorsal hippocampus. ROI-based analysis corroborated these findings (Figure S4). Statistically-significant reductions in bCBV were observed in the thalamus, dorsal hippocampus, nucleus accumbens, cingulate, prefrontal and somatosensory cortex of BTBR mice, while increased bCBV was observed in the hypothalamus and dorsal hippocampus (p<0.05, all regions, Student’s t-test followed by Hochberg’s correction). No statistically significant difference in the magnitude of mean arterial blood pressure between the two animal cohorts were observed (BTBR 99.6±2.0 mmHg; B6 100.6±1.2 mmHg, p = .67, Student’s t-test). The MABP values mapped were well within the cerebral blood flow autoregulation window within which alterations in blood pressure are homeostatically compensated by autoregulatory mechanisms and do not results in CBV alterations under comparable anaesthetic regimens [53], [54].

## Discussion

In-depth neurobehavioral characterizations have led to the identification of the BTBR mouse line as an experimental model for the investigation of neuro-biological determinants and potential treatments for autism-like symptoms. The robust behavioural phenotypes displayed by BTBR mice have prompted a rapid and widespread use of this mouse line in the preclinical community. Because neuro-behavioural traits are described and quantified with respect to reference populations, multiple investigators have examined and compared the behaviour of BTBR mice against that exhibited by B6 mice, a mouse line characterised by high sociability and low self-grooming [12]–[14], [17]–[20]. In an attempt to probe the translational relevance of this comparison for autism research, in the present work we used MRI to investigate anatomical and functional neuroimaging readouts that have been extensively used in patient populations. By directly comparing imaging readouts of pathological relevance in men and mice, an objectifiable assessment of the construct-validity of animal models of autism can now be obtained thus providing a translational bridge between clinical and preclinical research.

The main finding of the present work is the identification of robust and pervasive neuro-anatomical and functional alterations in BTBR mice with respect to B6. The presence of callosal agenesis and altered hippocampal commissure in BTBR mice has been already reported [19], [23], [24], [55]. Our results confirm and expand these findings, by providing for the first time a description of the three-dimensional topology of WM in this strain via DTI tractography. The approach also revealed major antero-posterior topological WM reorganisation in BTBR mice. The presence of large rostro-caudal WM bundles structures (Probst Bundles) was apparent in all BTBR subjects. Similar WM structures have been previously described in individuals with agenesis of the corpus callosum [56], [57] and in mouse models of altered chemotropic axonal guidance [58]. Tracts originating from the forceps major of the corpus callosum in BTBR also did not exhibit inter-hemispheric extension, but rather developed along the longitudinal axis of the brain. Intact inter-hemispheric tracts were observed in the anterior commissure, in ventro-medial thalamic areas and in a BTBR-specific WM formation recently described by Miller et al., [23], a finding that may be responsible for some degree of inter-hemispheric coherence described by the same authors.

While most idiopathic cases of ASD do not show callosal agenesis (or dysgenesis), diffusion MRI studies have consistently highlighted reduced callosal volume and fractional anisotropy in autistic patients with respect to control populations [59], [60]. In this respect, the lack of corpus callosum and presence of major topological WM alterations in BTBR mice could be interpreted as a potential extreme of the spectrum of callosal (and more generally WM) alterations observed in autistic patients and as such this neuroanatomical trait may be of potential translational relevance for autism research in the light of the recent under-connectivity theory of autism [61]. Consistent with this view, a growing literature documents social and linguistic deficits in patients with congenital agenesis of the corpus callosum [62]–[64] and very recent evidence suggests that as many as 10–30% of people with this condition would meet criteria for a diagnosis of an autism spectrum disorder [65], [66]. It should however be emphasized that callosal abnormalities have been described in association with more than fifty different metabolic and genetic disorders of the central nervous system including neuropsychiatric syndromes characterized by altered social competence like schizophrenia [67]. Moreover, surgical callostomy studies in epilepsy patients have demonstrated no adverse consequences in terms of personality and social behaviours, a finding that strongly argues against a distinctive or causal contribution of callosal disconnection to autism [68]. This notion has been recently confirmed by mouse studies showing unaltered sociability in B6 mice undergoing postnatal surgical lesion of the corpus callosum [19]. Collectively, the WM alterations observed in BTBR mice present analogies only with clinical subpopulations of autistic patients characterized by agenesis of the corpus callosum. The identification of mouse models exhibiting analogous phenotypes but intact inter-hemispheric alterations is warranted to elucidate the exact etiopathological contribution and assess the translational relevance of these findings.

VBM and cortical thickness measurements highlighted major and widespread GM alterations in BTBR affecting cortical regions but also subcortical substrates such as thalamus and dorsal hypothalamus. Whether these GM abnormalities mapped are independent of, or secondary to the WM rearrangement observed is not known. Our results are in good agreement with the findings of Ellegood and colleagues [24], which recently mapped GM volume in BTBR and B6 control mice using a similar MRI technique and observed analogous reductions in fronto-cortical areas and thalamus, and foci of increased GM volume in the hippocampus, cerebellum and olfactory bulbs. Importantly, the same work described analogous imaging alterations with respect to another sociable mouse strain (FVB/AntJ), thus corroborating the anatomical specificity of the morpho-anatomical changes in BTBR mice. An advantage of the present study is the use of complementary methods to map GM alterations, with cortical thickness analysis providing a direct and specific measure of cortical GM, and VBM offering an additional (but less specific) measure of GM volume across the whole brain that conflates information about morphology, size, and position [33]. Moreover, in contrast with the method employed by Ellegood and colleagues [24], our VBM data were generated upon the creation of study-specific reference template, a strategy that has been demonstrated to result in more accurate findings with respect to the use of prior reference templates [37]–[39]. The presence of converging regions of altered GM thickness and volume strengthens our confidence in the results and permits to rule out artefactual or unspecific contributions to the changes mapped. At the same time, the use of cortical thickness mapping greatly magnified inter-strain differences, revealing pervasive alterations in BTBR with respect to control that greatly exceed in size and distribution what previously observed by Ellegood and colleagues [24]. The augmented sensitivity of the cortical thickness method is consistent with the possibility to use less stringent family-wise error corrections in smaller cortical volumes with respect to larger whole brain VBM datasets.

Human mapping of GM volume and cortical thickness in autism have produced mixed results, a finding that probably reflects clinical and etiologic heterogeneity of the disorder as well as methodological differences. In spite of this, shared patterns of alterations are emerging. Increased fronto-cortical thickness and foci of reduced parietal GM have been described in frontal regions of adult autistic patients and children [69], [70]. Consistent findings have been recently reported as a result of a VBM meta-analysis of 24 multiple independent datasets [2]. The approach revealed the presence of decreases of GM volume in the bilateral amygdala-hippocampus complex and the bilateral parietal cortex and small focal GM increases in the middle-inferior frontal gyrus of adult autistic patients [2]. In the light of these findings, the large and prominent cortical thickness decrease and the widespread subcortical alterations observed in BTBR do not appear to be representative of typical clinical findings in autism. The fact that the only published reports of distributed cortical thinning in autism have been performed in sample populations including low-functioning autistic children (including mentally-retarded individuals) [71] or largely heterogeneous samples (including Asperger’s syndrome and other developmental disorders) [72] is of interest and should taken into account when the BTBR model is used for translational research. The presence of focal and robust increase in fronto-medial GM thickness and volume is consistent with recent clinical findings [70] and could be related to some of the social dysfunction exhibited by this strain [73].

fMRI mapping of CBV provides the first demonstration of widespread alterations in resting brain function in BTBR mice. The most prominent feature observed was the presence of reduced thalamo-cortical resting state CBV, an established marker of basal metabolism [25], [27], [49]. This results is in keeping with (and could conceivably be a causative factor of) the reduced cortical thickness observed in BTBR, as a number of imaging studies have consistently demonstrated a linear relationship between functional activity and cortical GM thickness [74]. This finding is also in good agreement with previous studies in autistic patients using metabolic or haemodynamic correlates of resting neuronal function. Reduced metabolism in the thalamus, fronto-cortical regions and ventral (but not dorsal) striatum have been described in adult patients [7], [75]. Similarly, robust and widespread reduction in cortical perfusion have been observed in young [5], [6], [76]–[79] and adult [78] autistic patients, with a predominant involvement of the prefrontal, cingulate, insular and temporal cortex. The presence of fronto-cortical and somatosensory hypo-perfusion in BTBR mice is thus transitionally relevant, as it recapitulates a common clinical neuroimaging finding in autism and defines a research model that might be employed to investigation the cellular and neurobiological mechanism that govern this phenomenon and its relationship with behavioural symptoms of the disorder.

In conclusion, we describe neuroimaging evidence of prominent neuro-anatomical and functional alterations in the brain of BTBR mice. The major topological WM reorganization observed, together with the presence of widespread cortical and subcortical GM alterations do not appear to be representative of the neuroanatomical alterations typically observed in autistic patients. The presence of reduced fronto-cortical metabolism is of potential translational relevance, as this feature recapitulates previously-reported clinical observation. Our data underscores the use of translational neuroimaging readouts to obtain an objectifiable assessment of the construct validity of mouse models of autism.

## Supporting Information

Figure S1 **Major FA reduction in dorsal hippocampal commissure and corpus callosum of BTBR mice.** (a) Location of regions of interest (ROIs) used for quantitative FA analysis. The regions are overlaid onto the corresponding reference (B6) FA template. (b) Mean FA in representative ROIs. Data are expressed as means ± SEM. **p<0.01; ***p<0.001 vs. B6 controls, one-way ANOVA followed by Hochberg’s correction for multiple comparisons. [DHc: dorsal hippocampal commissure; ec: external capsula; CC: corpus callosum; aca: anterior commissure; Fmi: forceps minor of the corpus callosum].
(TIF)

Figure S2 **Novel cortico-cortical inter-hemispheric tracts in BTBR mice.** Diffusion tensor tractography of representative cortico-cortical inter-hemispheric tracts in BTBR (bottom) mice obtain by placing seed areas in the BTBR-specific white matter formation recently described by Miller et al (2013). Seed regions used for tractography are displayed in green/red. No such tracts were found in analogous anatomical areas of control B6 subjects (top), where antero-posterior striato-thalamic connections were instead found.
(TIF)

Figure S3 **Widespread GM alterations in BTBR mice.** (a) Location of region of interest (ROI) used for quantitative analysis of gray matter volume (GMV). The regions are overlaid onto the corresponding reference (B6) anatomical template. (b) Mean GMV in representative ROIs. Data are expressed as means ± SEM. **p<0.01; ***p<0.001 vs. B6 controls, one-way ANOVA followed by Hochberg’s correction for multiple comparisons. [Amy: amygdala; DHyp: dorsal hypothalamus; LTh: lateral thalamus; Hc: dorsal hippocampus; mPFC: medial prefrontal cortex; dPFC: dorsal prefrontal cortex; Ins: insular cortex].
(TIF)

Figure S4 **Widespread bCBV alterations in BTBR mice.** (a) Location of region of interest (ROI) used for quantitative analysis of bCBV. The regions are overlaid onto a corresponding (B6) reference anatomical template. (b) Mean bCBV in representative ROIs. Data are expressed as means ± SEM. *p<0.05; **p<0.01; ***p<0.001 vs. B6 controls, one-way ANOVA followed by Hochberg’s correction for multiple comparisons. [Hyp: hypothalamus; Th: thalamus; dHc: dorsal hippocampus; Cpu; caudate putamen; Acb; nucleus accumbens; Ins: insular cortex; mPFC: medial prefrontal cortex; Cg: cingulate cortex; SS: somatosensory cortex].
(TIF)
