# Supplementary figures and images for: Neuroimaging Evidence of Major Morpho-Anatomical and Functional Abnormalities in the BTBR T+TF/J Mouse Model of Autism
Source: PLoS One. 2013 Oct 16;8(10):e76655. doi: 10.1371/journal.pone.0076655 (PMC3797833; doi:10.1371/journal.pone.0076655)

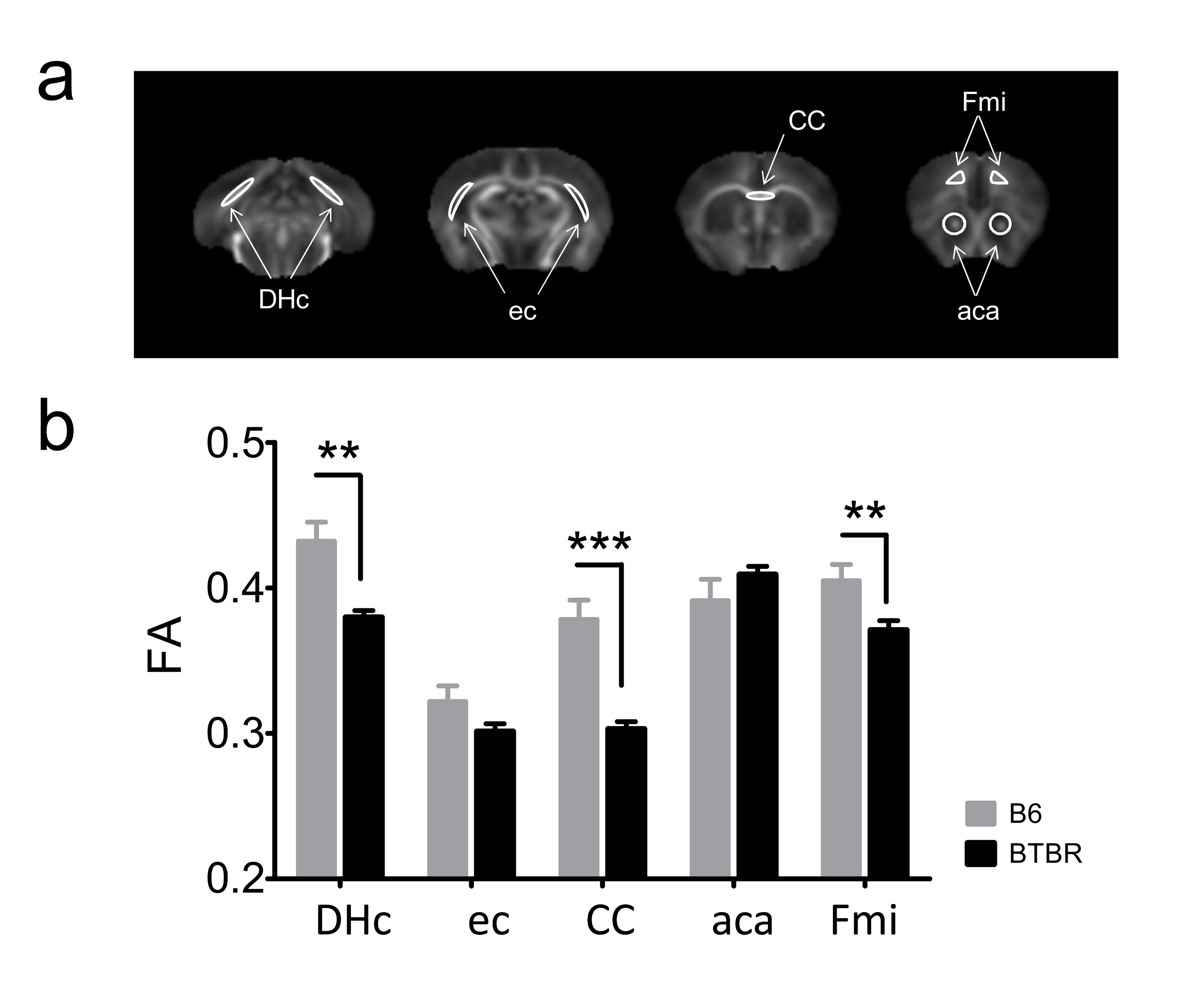

Supplement: Figure S1 — Major FA reduction in dorsal hippocampal commissure and corpus callosum of BTBR mice. (a) Location of regions of interest (ROIs) used for quantitative FA analysis. The regions are overlaid onto the corresponding reference (B6) FA template. (b) Mean FA in representative ROIs. Data are expressed as means ± SEM. **p<0.01; ***p<0.001 vs. B6 controls, one-way ANOVA followed by Hochberg’s correction for multiple comparisons. [DHc: dorsal hippocampal commissure; ec: external capsula; CC: corpus callosum; aca: anterior commissure; Fmi: forceps minor of the corpus callosum]. (TIF) [file pone.0076655.s001.tif]

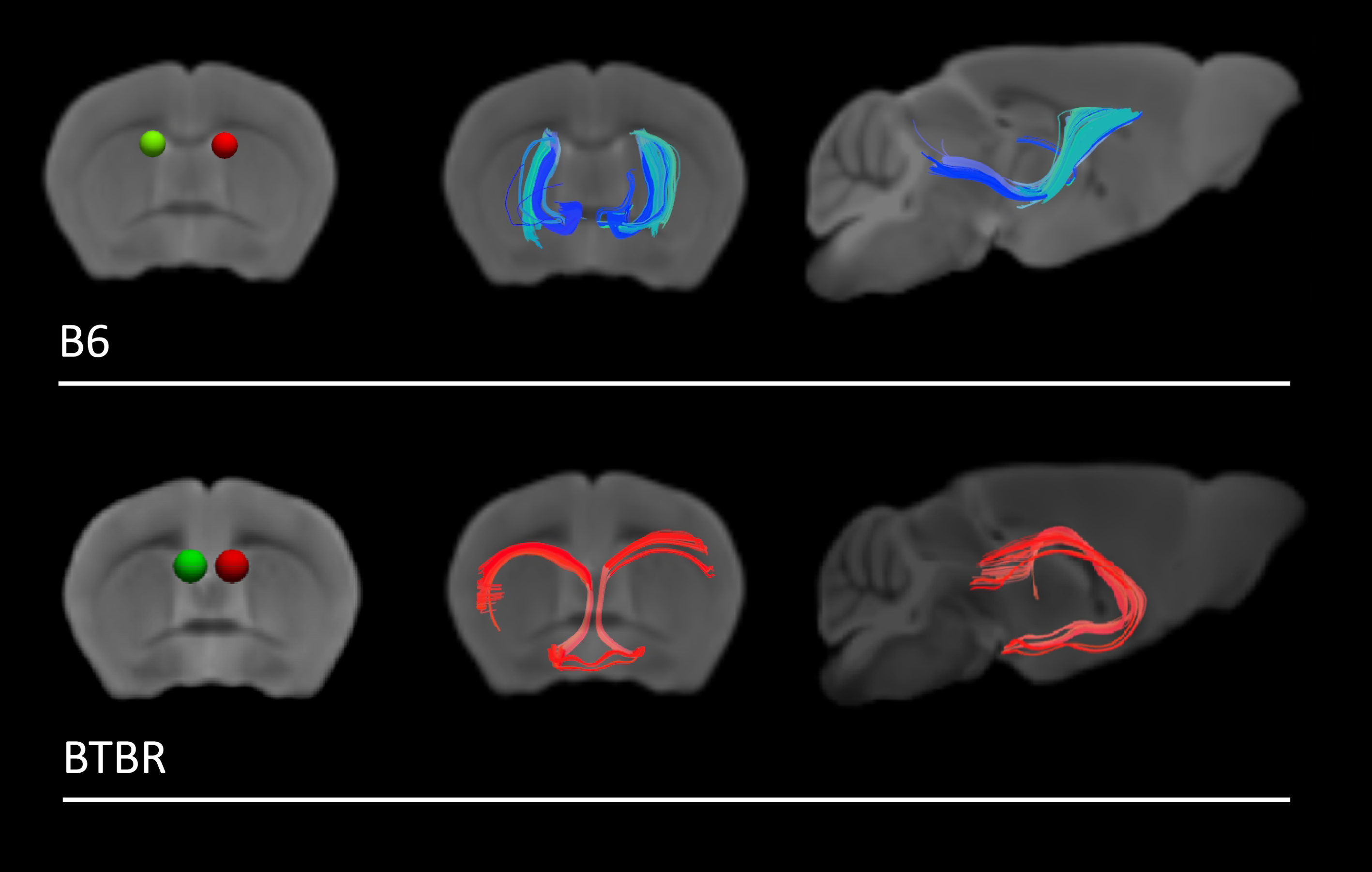

Supplement: Figure S2 — Novel cortico-cortical inter-hemispheric tracts in BTBR mice. Diffusion tensor tractography of representative cortico-cortical inter-hemispheric tracts in BTBR (bottom) mice obtain by placing seed areas in the BTBR-specific white matter formation recently described by Miller et al (2013). Seed regions used for tractography are displayed in green/red. No such tracts were found in analogous anatomical areas of control B6 subjects (top), where antero-posterior striato-thalamic connections were instead found. (TIF) [file pone.0076655.s002.tif]

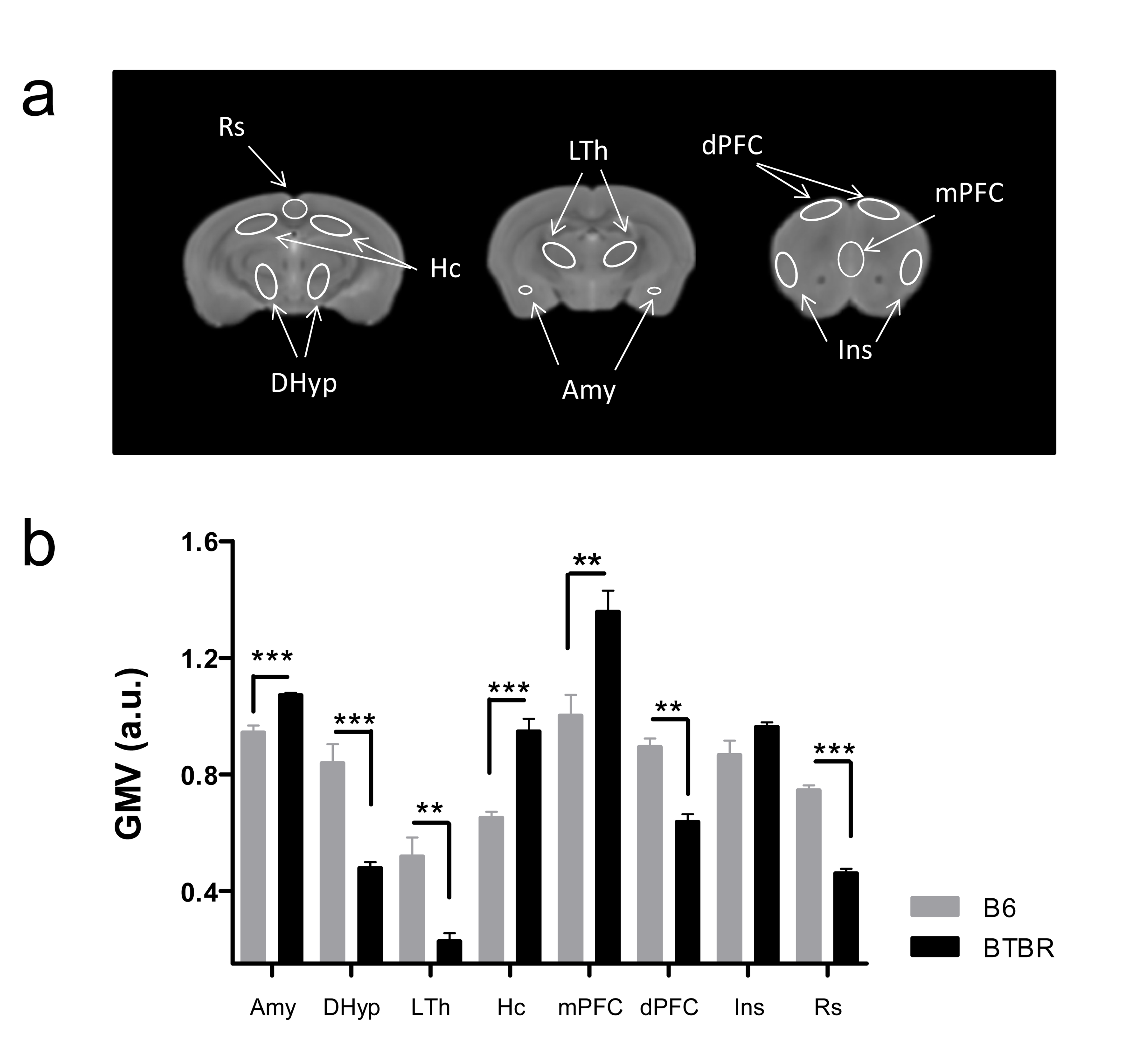

Supplement: Figure S3 — Widespread GM alterations in BTBR mice. (a) Location of region of interest (ROI) used for quantitative analysis of gray matter volume (GMV). The regions are overlaid onto the corresponding reference (B6) anatomical template. (b) Mean GMV in representative ROIs. Data are expressed as means ± SEM. **p<0.01; ***p<0.001 vs. B6 controls, one-way ANOVA followed by Hochberg’s correction for multiple comparisons. [Amy: amygdala; DHyp: dorsal hypothalamus; LTh: lateral thalamus; Hc: dorsal hippocampus; mPFC: medial prefrontal cortex; dPFC: dorsal prefrontal cortex; Ins: insular cortex]. (TIF) [file pone.0076655.s003.tif]

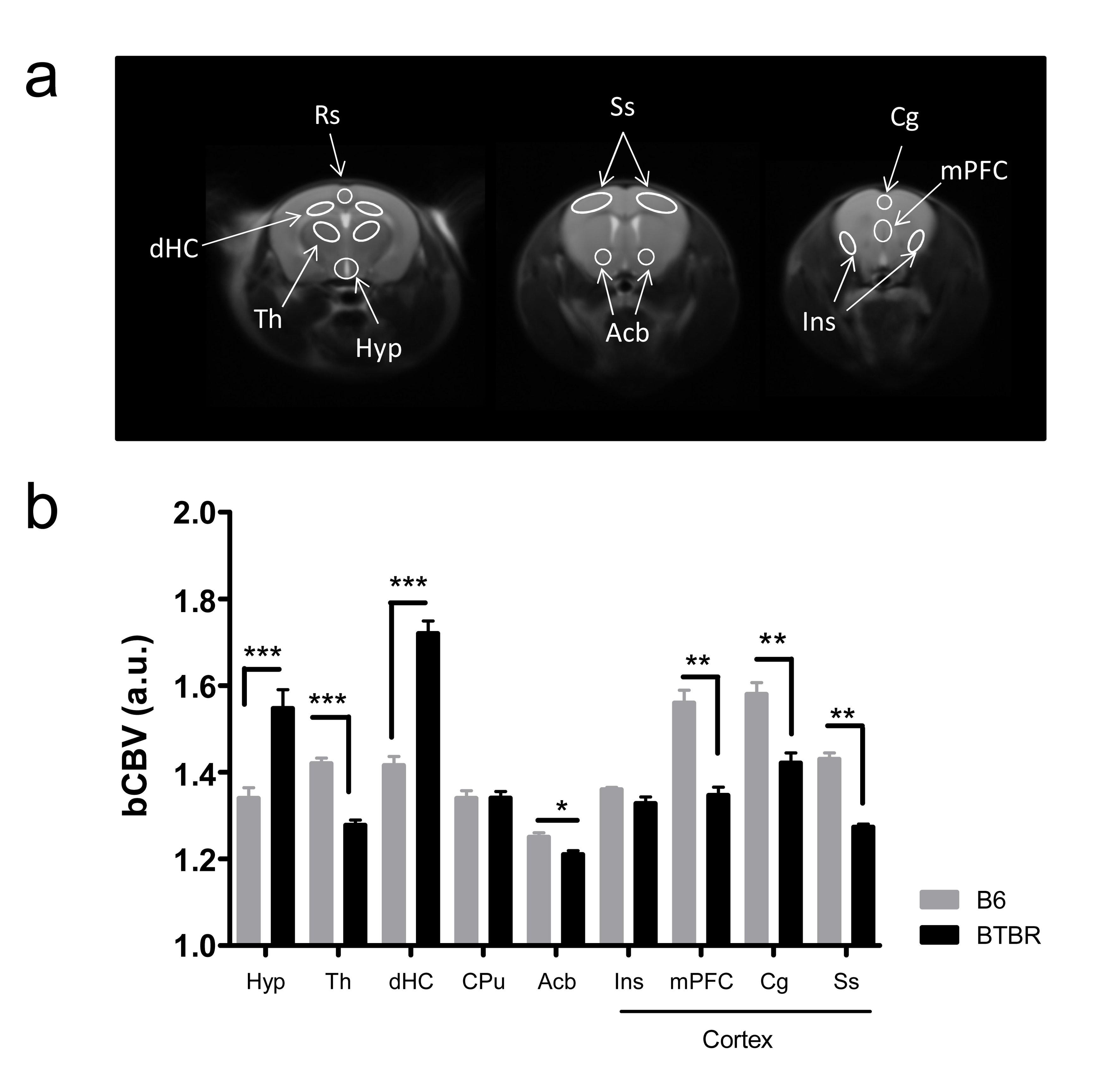

Supplement: Figure S4 — Widespread bCBV alterations in BTBR mice. (a) Location of region of interest (ROI) used for quantitative analysis of bCBV. The regions are overlaid onto a corresponding (B6) reference anatomical template. (b) Mean bCBV in representative ROIs. Data are expressed as means ± SEM. *p<0.05; **p<0.01; ***p<0.001 vs. B6 controls, one-way ANOVA followed by Hochberg’s correction for multiple comparisons. [Hyp: hypothalamus; Th: thalamus; dHc: dorsal hippocampus; Cpu; caudate putamen; Acb; nucleus accumbens; Ins: insular cortex; mPFC: medial prefrontal cortex; Cg: cingulate cortex; SS: somatosensory cortex]. (TIF) [file pone.0076655.s004.tif]
